# Integrated care networks in multidisciplinary rehabilitation therapy services for childhood oncology close to home: lessons learned from an international environmental scan

**DOI:** 10.1007/s00520-025-09421-w

**Published:** 2025-04-23

**Authors:** L. B. Kleinlugtenbelt, J. W. Gorter, E. C. van Dalen, M. Ketelaar, W. J. E. Tissing

**Affiliations:** 1 Princess Máxima Center for Pediatric Oncology, Utrecht, The Netherlands; 2 Department of Rehabilitation, Physical Therapy Science and Sports, UMC Utrecht Brain Center, University Medical Center Utrecht, Utrecht, The Netherlands; 3 Center of Excellence for Rehabilitation Medicine, UMC Utrecht Brain Center, University Medical Center Utrecht, and de Hoogstraat Rehabilitation, Utrecht, the Netherlands; 4 Department of Pediatric Oncology, University of Groningen, University Medical Center Groningen, Groningen, the Netherlands

**Keywords:** Childhood oncology, Integrated care networks, Continuity of care, Quality of life, Multidisciplinary rehabilitation therapy services, Allied healthcare professionals, Psychologists

## Abstract

**Background:**

Integrated care networks (ICNs) close to home have the potential to improve continuity and quality of care for children with cancer and their families during and after treatment. Our goal is to develop such a network for multidisciplinary rehabilitation therapy services (RTS) in The Netherlands, but we lacked a good understanding of an ICN and the factors to make it successful.

**Purpose:**

The aim of the study was to learn from initiatives in ICN’s internationally, how are ICN’s developed, how does it promote collaboration and what are facilitators and barriers in its development and use?

**Methods:**

We performed an environmental scan. First, we performed a systematic literature search (PubMed) focussing on ICNs for childhood oncology. Secondly, we sent a survey regarding development and use of ICNs to international childhood cancer centers. Participating centers were asked to share information about their initiatives in providing care close to home. Data were summarized descriptively and analyzed using content analysis.

**Results:**

The literature search did not reveal any relevant publications. The results from the survey, including15 countries, provided valuable insights in the understanding of a good ICN, its facilitators and barriers, and the potential added value of developing ICNs close to home to provide continuity and quality of care.

**Conclusions:**

Our study highlights the perceived importance of ICNs for multidisciplinary RTS in pediatric oncology and provides valuable information for the formation of such a network. Information about the needs from the perspectives of children and parents is currently missing and essential to develop successful ICNs.

**Supplementary Information:**

The online version contains supplementary material available at 10.1007/s00520-025-09421-w.

## Introduction

Around 600 children are diagnosed with cancer each year in the Netherlands, with currently more than 16.000 survivors [1]. The 5-year survival rate after childhood cancer is 80% in high income countries [2]. However, many patients suffer from side effects during and shortly after treatment and 75% of long-term survivors have 1 or more late adverse effects of the treatment. With higher cure rates, the total number of childhood cancer survivors increases, resulting in increasing numbers of patients with long-term medical, physical, and psychosocial late effects and needs, often extending into adulthood [3, 4]. Both the early and late adverse effects need the right care in the right place at the right time.

Since 2018, care for children with cancer in The Netherlands is centralized in the Princess Máxima Center for pediatric oncology in Utrecht. The mission is to cure every child with cancer with optimal quality of life. We aim to centralize care when needed and to provide care locally when possible. Continuity of care closer to home is realized by administering parts of the oncological care in one of 15 satellite hospitals. Improving optimal quality of life is getting more important, beside the mission to cure every child with cancer. For this, multidisciplinary rehabilitation therapy services (RTS) help children and survivors return to daily life and daily functioning or improve skills that have been lost or impaired because of the cancer treatment. They include services provided by physiotherapists, psychologists, dieticians, occupational therapists, speech and language therapists, and are preferably delivered even closer to home and in the community setting, because of less travel time and better participation rates when in the patients’ own environment.

In a previous survey we found that local PPTs in the Netherlands only see none to four children with cancer in their whole career [5]. This, in combination with the diversity of cancer types, developmental stages (0–18 years) and various needs, results in a lack of specialised knowledge and experience for most PPTs in the community.

Thus, we need to improve continuity and quality of this care by better organization of its transition and improve collaboration between professionals.

Developing an integrated care network (ICN) for multidisciplinary RTS could be a solution to overcome the above-mentioned care and knowledge gaps and may contribute to the continuity and quality of care for children with cancer during and after treatment. Such a network would allow for sharing knowledge, developing skills, and improving accessibility and communication [5]. In this study, the definition of ICNs is based on the Rainbow Model of Integrated Care (RMIC) and defined as a coordinated way of working across multiple professionals, organisations and sectors in order to improve the health, quality of care and economic outcomes for a targeted (sub)population [6–9].

Worldwide, there is an understanding that the concept of network medicine is critical to meet the needs of children with cancer during and after treatment [4]. Facilitating the right care in the right place is one of the most important objectives of network care. For our goal we aim to develop an ICN, including healthcare close to home, because this approach helps to prevent more expensive care in specialized centers, but also to move care closer to people’s homes, or at home, whenever possible, for better quality of life and efficiency of care [10–12]. Goals of such healthcare networks are often related to a quadruple aim, i.e., improving the patient experience of care, the health of populations, reducing per capita costs and improving the work life of those who deliver care, the professionals experiences [13].

Ideally, the development of an ICN can build on existing knowledge and experience in the field. Environmental scans (ES) are one way to identify and collate a large body of information seeking to achieve this objective [14, 15]. We performed a literature search and an international ES in order to identify and learn from existing ICNs for multidisciplinary RTS to improve the care of pediatric oncology patients during and after treatment close to home, with the aim to learn from initiatives in ICN’s internationally, how ICN’s are developed, how they stimulate collaboration and what the facilitators and barriers are in developing and collaboration?

## Methods

In this study, we performed an environmental scan (ES) using a two-step process. In part 1) we systematically searched for the available evidence in the literature reporting on ICNs in pediatric oncology. We focussed on the following topics: 1) elements of developing and/or collaborating in ICNs on the micro (patients), meso- (professionals and organizations), and/or macro- (system, i.e., legislation and regulations) level of the RMIC [6–9], and 2) indicators of the quadruple aim, i.e., improving the health of populations, improving the patient and caregiver experience, reducing the per capita cost of health care and improving the work life of providers [13], 3) determinants of efficacy (process determinants (e.g. trust) structural determinants (e.g., ties between actors), and contextual determinants (e.g. the institutional environment)) [16, 17]. In part 2), we collected user experiences using an international survey, sent to international childhood cancer centers across the world, regarding ICNs for childhood oncology and their facilitators and barriers, in order to learn from other initiatives. Data was summarized descriptively.

### Part 1: A systematic literature search

#### Search methods for identification of studies

We searched PubMed/MEDLINE (from 1945 to 7 September 2023). The search strategy (using a combination of controlled vocabulary and text words) is shown in the supplementary data. In addition, information about studies not registered in PubMed/MEDLINE was identified by searching the reference lists of included studies and review articles.

#### In- and exclusion criteria

Studies published in English were considered eligible for inclusion if they focused on (evaluation of) ICNs concerning multidisciplinary RTS for childhood cancer patients and/or survivors. We defined ICNs as a coordinated way of working across multiple professionals, organisations and sectors in order to improve patient care. There were no restrictions for type of cancer, as long as they focused on patients (during or after anti-cancer treatment) diagnosed with cancer at age <  = 21 years. Studies of research networks (defined as networks intended solely to support research to improve care), papers on only a reported vision about care networks, or only reporting on knowledge sharing and not about collaboration within the care, were excluded. If not all participants were eligible for inclusion (e.g., not only children with cancer) a publication was only included if separate data on eligible participants are available.

#### Selection of studies

**Fig. 1:**
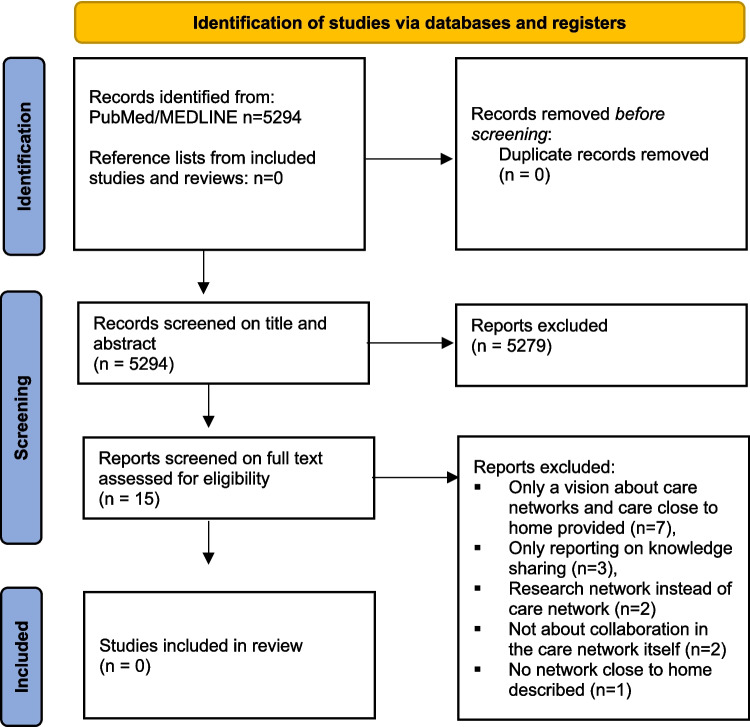
Flow diagram of the selection of studies. From: Page MJ, McKenzie JE, Bossuyt PM, Boutron I, Hoffmann TC, Mulrow CD, et al. The PRISMA 2020 statement: an updated guideline for reporting systematic reviews. BMJ 2021;372:n71. 10.1136/bmj.n71

After the search strategy was employed, all identified titles and abstracts were screened for eligibility by two independent reviewers. They obtained a full-text version of each study that appeared to meet the inclusion criteria based on the title or abstract, or both, or studies for which this was unclear, and further assessed these for inclusion. Any discrepancies between reviewers were resolved by consensus. A flow diagram of the complete selection of studies in the search is included (Fig. 1).

#### Data extraction, risk of bias assessment, and data synthesis

Since no eligible studies were identified, data extraction and risk of bias assessment by two independent authors using a standardised form and data analysis were not applicable.

### Part 2: An international survey

#### Procedure

A survey, developed and discussed by the research team, was distributed to pediatric oncology centers around the world (supplementary data II). The survey questions were focused on information about their experiences and challenges in providing care close to home.

A personal e-mail was sent to personal contacts (pediatric oncologists) in pediatric oncology centers across the world with the opportunity to forward the email to a colleague with more experience in the field of ICNs. The e-mail included background information on the topic and a definition of ICN (supplementary data II). The respondent was asked to answer the questions of the survey as a representative of the center. By snowball sampling we finally sent out the survey to 26 pediatric oncology centers in 19 different countries. In case of no response a reminder was sent two weeks after the first deadline.

In the survey we asked closed-ended and open-ended questions about current organization, experiences, and initiatives in coordinating the care for children with cancer and survivors close to home. Topics included the added value of an ICN for care close to home during and after treatment, barriers and facilitators to create an ICN and how an ideal ICN should look like (supplementary data II, for the complete survey).

#### Data analysis

Data of the survey responses were transferred into an excel sheet. Responses of closed-ended questions were analyzed using descriptive statistics. The open-ended questions related to current initiatives, the perceived added value, perceptions on the ideal integrated care network, and the questions focusing on facilitators and barriers were analyzed qualitatively by the first author, based on content analysis [18–21]. For each of these questions, relevant parts in the responses were selected and coded. Codes were then grouped into key categories to capture the essence of the responses, and content labels were assigned to these categories. The content labels were further discussed and refined within the research team.

## Results

### Part 1: A systematic literature search

The search of PubMed/MEDLINE identified 5294 references. After screening the titles/abstracts 5279 references clearly did not meet the inclusion criteria. For 15 references, full texts were screened, but again none was eligible for inclusion. Thus, no papers were included in the literature search (Fig. 1).

### Part 2: An international survey

#### Demographics

In total, 22 persons from 22 hospitals from 15 different countries responded: Europe (Belgium, Denmark, France, Germany, Ireland, Italy, Slovakia, Spain, Sweden, The Czech Republic, UK), North America (Canada, USA) and Australasia (Australia, Indonesia). No response from Austria, Brazil, Chile, and Swiss. The response rate was 85%. (Table 1, additional demographic data).

**Table 1:** Demographics participants survey

Survey demographics	N (%)	
**Response on survey** yes no	22 (85%) 4 (15%)	
**Disciplines of respondents** Pediatric oncologist/medical doctor/pediatrician/fellow Physiotherapist Researcher (lecture/professor/PhD candidate) Psychologist Neuro psychomotor therapist Rehabilitation clinic scientific director Pediatric Physical Medicine and Rehabilitation Physician	5 (23%) 9 (41%) 4 (18%) 1 (4,5%) 1 (4,5%) 1 (4,5%) 1 (4,5%)	
**Countries responded (total)** In Europe In North Ameri**ca** In Australasia	15 (100%) 11 (74%) 2 (13%) 2 (13%)	
**Range in which the center/hospital treats children** 0–100 km 100–250 km 250–500 km > 500 km	6 (27%) 8 (36%) 2 (10%) 6 (27%)	
**Distance next center/hospital** 0–100 km 100–250 km 250–500 km > 500 km	4 (18%) 6 (28%) 4 (18%) 8 (36%)	
**Collaboration agreement with other centers/hospitals closer to home?** yes no	15 (68%) 7 (32%)	
**New patients a year** 0–100 100–250 250–500 > 500	4 (18%) 11 (50%) 5 (22%) 2 (10)	
**Allied healthcare professionals in clinic** Psychologist Pediatric physical therapist Dietician Occupational therapist Speech and language therapist Other mentioned	22 22 21 20 21	Music/play/art therapists, orthotists, prosthetists, palliative care team, radiographers, social work, neuropsychologists, psychometrist therapist, child life specialist, caregivers providing reflexology and sophrology, adapted physical activity teacher, exercise therapist, pharmacists, social healthcare workers, nurse consultants and trial coordinators
**Outpatient clinic for follow-up** Yes no	20 (90%) 2 (10%)	
**Integrated care network (ICN)** Yes no	7 (32%) 15 (68%)	
**ICN of added value** Yes no	22 (100%) 0	

#### Initiatives in integrated care close to home

Seven of the 15 countries, Canada, France, Germany, Indonesia, Italy, the UK, and the USA reported to have a certain form of ICN by referral. The initiatives of these seven countries were quite different per country and the initiatives in ICNs were not structurally evaluated. Responses of these seven countries have been summarized descriptively below per country.

In the UK, there are ICNs on individual referral basis. They have an outreach service which will do initial joint sessions with local teams for handovers of more complex patients. Each region has their own set-up. Their network activities have not been evaluated.

In Canada, health care is province/territory specific. For example, there are satellite hospitals available for assessment and treatment and there are Pediatric Rehabilitation Services for the province of Alberta. Recently, the Canadian Pediatric Oncology Physiotherapy group started to connect physiotherapists across the country. The Pediatric Oncology Group of Ontario (POGO)’s Provincial Pediatric Oncology Satellite Program transfers aspects of a child’s care, for eligible patients, to a community hospital closer to home across Ontario [22]. The POGO Satellite Program and other initiatives have not been formally evaluated but they do check the quality annually.

The respondent from the state of Minnesota (USA) mentioned to have cooperation with six rehabilitation clinics and to have an Affiliate Program Office [23]. There are no evaluations available. St Jude Children’s Research Hospital (Tennessee) has an “oncology transition in care program” that makes referrals and helps families get connected to services closer to their homes. They are in a unique position because patients come to St. Jude from across the US and around the world. This makes it very challenging to develop an ICN.

In Piedmont Italy, there is a pediatric oncology network that comprehends a regional reference center and collaboration with 10 satellite hospitals in the surrounding area [24]. The network has not been formally evaluated.

In Germany, integrated care is not organized in a centralized way. Integrated home-based care does not exist yet in all disciplines of care and is just starting. There are specialized palliative care (PC) teams integrated in the clinic and they provide multi professional care at home. For physiotherapy and exercise therapy, patients and survivors are integrated into structures close to home on an individual basis, by Network ActiveOncoKids [25–27]. The network has not been formally evaluated.

In France, pediatric physical therapy is provided by private physiotherapists, and other integrated care can be done in this context as well. The French Health care system or private health mutually pay part of the cost of psychological care [28]. Only for childhood cancer survivors, additional financial help is provided by the French Health care system. For dietary management, there is a network of private dieticians specifically trained in hemato-oncology who have agreed to see patients at a fixed rate. Over the last 3 years, the regional health agency has introduced a package for patients who have completed their treatment, enabling them to benefit from a psychological assessment, and/or an adapted physical activity assessment, and/or a dietary assessment close to home. There’s no evaluation available.

Indonesia mentioned to have integrated care by referral to the Physical Medicine and Rehabilitation department. An integrated e-referral system is being developed but not working well yet. They have preferred partners for rehabilitation (for Prosthesis, Orthosis, Exercise, Physical modalities or Electrodiagnosis/Electromyography). There’s no website or evaluation available.

#### The added value of ICNs for multidisciplinary RTS and how integrated care should ideally look like

All respondents agreed that there is an added value of creating and collaborating within an ICN to improve care close to home. Respondents expect it to enhance communication between healthcare professionals, to improve knowledge and experience among healthcare providers, to increase quality and continuity of care, it increases care quality and continuity, providing better support from diagnosis to survivorship. Moreover, respondents expect it to offer resources/finance/capacity, it offers more resources and reduces the workload for oncology teams, it improves quality of life, it improves the quality of life and mental well-being of the child and family, it encourages participation in social activities, reducing separation from home and community, it reduces distances, travel time and it decreases hospitalizations and health inequalities.

In the descriptions of respondent what an ideal ICN should look like, the following content labels/topics emerged: Communication: Digital consultations and communication pathways are essential, along with financial coverage to avoid additional strain on families. Resources/finance/capacity: All necessary healthcare professionals should be involved, with regular briefings and easy referral pathways to ensure coordinated care. Distances: A head multidisciplinary team should communicate with smaller centers, covering the whole country. Other: Services should be accessible in locations convenient for families, with a base center coordinating training and treatment across the network (more details can be found in Table 2).

**Table 2:** Perspectives of participants on integrated care networks

	What is the added value of integrated care networks to improve the care in pediatric oncology close to home?	What should an ideal integrated care network close to home look like?
**Communication**	Better communication between health care professionals	Digital, regular and central consultations available/multidisciplinary meeting forum Communication pathways and educational pathways and referral pathways Professionals fully informed about the child’s health condition and his/her needs Medical record sharing system
**Knowledge**	Improve capabilities in knowledge and experiences of health care professionals (knowledge gain)	Joint training sessions Involve all the necessary healthcare figures and include a program for transition to adulthood and a case manager for survivors with chronic disabilities
**Quality and continuity of care**	Better quality of care, better continuity of care More time (frequency) for the patient close to home by the local health care professional Better support for patient from acute phase to survivor phase	Guarantee continuum of care from diagnosis to reintegration to the community with minimal impact from the treatment (long term side effects) for patient and family
**Resources/finance/capacity**	Greater choice of offers, conservation and use if resources Decrease of workload for oncology teams by outsourcing certain aspects to the care network for better focus on other important things	Skilled workforce Financially covered Minimal waiting times All professionals should be involved (physician, nurse practitioner, physiotherapy, occupational therapy, speech therapist, audiologist, social worker, kinesiologist, psychologist, teachers, child life specialist) Access services for children/families close to home
**Quality of life**	Improve quality of life of the child/family Improve mental well-being	
**Participation**	Improve participation in social community activities and seeing their friends/decreased separation from home and community	
**Distances**	Reduction of travel time	Short distances availability and cover the whole country
**Other**	Less hospitalization Reduce health inequalities Reduces burden in acute services Less loss of income	Outreach from the main treating hospital to build the network, base center responsible for training and coordination It should be structured (it often relies mainly on the availability and good heartedness of each involved profession)

#### Barriers and facilitators for multidisciplinary RTS in creating an ICN close to home

All responders mentioned barriers and facilitators in developing, collaborating and implementing an ICN close to home. Table 3 summarizes them.

**Table 3:** Barriers and facilitators in creating an integrated care network close to home

	What are barriers to develop and collaborate in an integrated care network?	What are facilitators to develop and collaborate in an integrated care network?
Trust	Lack of Trust from patients/families and local teams Local therapists are afraid to provide care Fears of contact Fears for palliative care, everyday stress of families Unclear responsibilities	Relationship building Assure families that every team member to commit to the best possible outcomes for their child and will utilize all resources available to support
Communication	Trouble with communication Unclear set-up and contact	Initial joint sessions for more complex patients Online counselling, meetings in multidisciplinary team Case management Exchange of experience, discussion and collaboration Encourage to contact each other, also in follow-up
Knowledge	Lack of knowledge and experiences in childhood oncology Lack of guidelines and clear recommendations	Training, education and up-to-date knowledge (more frequent complications during treatment, adverse/side effects of oncology treatment, red flags and indications for therapy and therapeutic approaches) Guidelines and recommendations Child specific needs
Resources/Finance/Capacity	Lack of capacity of health care professionals Lack of resources for local health care professionals to gain knowledge Lack of financial support from the government vs private funding Health insurance system (not all care is covered and not affordable for most families) Lack of supportive carefree of charge during treatment	More capacity in trained health care professionals Subsidized companies by public health system Benefit patients with health care package (France) Government support
Distances	Significant distances	
Other	Lack of motivation of local health care professionals	

Barriers: Trust, lack of trust from patients, families, and local teams; Uncertainties and fears of healthcare providers regarding care provision, especially for palliative care; Communication, issues with communication and unclear contact points and responsibilities; Knowledge, lack of knowledge and experience in childhood oncology, as well as a lack of guidelines and recommendations; Resources/Finance/Capacity, shortage of healthcare professionals, insufficient resources for training, and a lack of financial support from the government; Distances, significant physical distances pose a challenge and Other, lack of motivation among local healthcare professionals.

Facilitators: Trust, building relationships and ensuring that every team member is committed to achieving the best outcomes for the child; Communication, joint sessions for complex cases, online counseling, case management, and encouraging collaboration and experience sharing; Knowledge, training, guidelines, and knowledge of child-specific needs; Resources/Finance/Capacity, increased capacity of trained healthcare professionals, government subsidies, and health insurance packages that cover care costs.

## Discussion

It has been widely recognized that it is important to administer care close to home for children and their families during and after treatment for childhood cancer [29–34]. We conducted the first international ES with the aim to identify and learn from existing ICNs for multidisciplinary RTS close to home to improve the care of pediatric oncology patients during and after treatment.

The main finding of our ES is twofold. First, we did not find any publications on developing and collaborating within ICNs for RTS in childhood oncology. We only found initiatives in sharing knowledge with the community [30–32] and research networks instead of care networks are informative [35].

Second, the results from the survey gave us valuable insights in the vision of healthcare providers from Western Europe, North America, and Australasia on this type of ICN, their initiatives in ICNs, and experienced barriers and facilitators. Currently, there is a worldwide lack of published existing programming and of evaluated ICNs for RTS.

All respondents agreed on the added value of ICNs for RTS to provide care closer to home for children and their families during and after treatment for childhood cancer. Themes like finding each other, connecting with each other and entrusting each other in referring care was considered to be very important. Furthermore, getting insights in the needs of children with cancer and their families, training, education, up-to-date knowledge and developing and sharing guidelines can contribute to reach this aim. Integrated care seems to be of added value because it might lead to minimalization of hospitalizations, less burden on acute services and a decrease in the workload of oncology teams by outsourcing certain aspects to the ICN with the benefit to be able to better focus on other important goals such as the complex patient care, research, or guidelines development. Respondents mentioned the importance of having equality in the availability of care for every child with cancer and their families. Also, it seemed important to pursuing the same goal, aim and vision within the ICN to be successful in collaborating to improve continuity and quality for care.

Lack of capacity, finance, and resources are reported as barriers in developing and collaborating in ICNs. More capacity in trained health care professionals and government support are needed to facilitate the development of, and collaboration in, ICNs in RTS for childhood oncology.

Although no publications on ICN for RTS in pediatric oncology patients were available, there is literature available from other fields in network care that highlight which issues are important to be successful in building and implementing ICNs [16, 17], which are mostly in line with the results of our survey. For example trust, working together and being able to refer care from hospital to community and back, but also the need of trust from children and families in the transition of care from hospital to care close to home. Clear communication, clarity about responsibilities, and knowledge sharing between professionals are also important, as is having a shared vision and goals within the network [16, 17], and knowing what the needs and perspectives of the child and families are.

Our study has several strengths. We were successful in obtaining a broad international perspective on ICN from RTS for children and their families during and after treatment for childhood cancer close to home.

A limitation might be that we did not include information on networks for other childhood diseases or adult ICNs. However, a main characteristic of pediatric oncology is that it often is provided through one or only very few specialized hospitals or centers in a large geographical area. Childhood cancer has a relatively low incidence rate as compared to other childhood chronic conditions such as cerebral palsy or muscular diseases. So, the design of an ICN for childhood oncology likely would be different than those for more common diseases. The comparison with adult cancer care differs with respect to different cancer types and treatment, with different adverse effects, and a larger amount of center and hospitals across the countries. Secondly, we did not include information about the needs from the perspectives of children and parents in care close to home. Thirdly, the survey was sent to personal contacts (pediatric oncologists) at centers around the world, with the opportunity to forward the email to a colleague with more experience in the field of ICNs. We were not able to reach all centers/countries worldwide. In particular responses from low- and middle-income countries are limited. Another limitation of the study is the relatively low number of respondents. While encouraged everyone to forward it to other centers, we were not able to reach all centers/countries worldwide. In particular, responses from low- and middle-income countries are limited. Although this study was a small survey of 22 centers across the world, we found a lot of similar answers in the survey, so we think these findings still provide us with important lessons to draw conclusions [36]. A limitation is that majority of the responders were from European counties only. Lastly, four of the in total 26 centers (15%) did not respond.

### Our future plans and vision

Our vision to develop an ICN for multidisciplinary RTS for pediatric oncology is further strengthened by this ES. Such a network would allow for sharing knowledge, developing skills, and improving accessibility and communication. We will use our findings in developing such an ICN in the Netherlands taking into account the reported barriers and facilitators. Moreover, we are planning to gain more in-depth information about the experiences and needs of children with childhood cancer and their families with care close to home to inform the development of an ICNs.

## Conclusion

This ES highlights the gap in literature about ICNs for multidisciplinary RTS in pediatric oncology. The survey gave insights in the facilitators and barriers, and the potential added value of developing such ICNs. Further in-dept information about the needs from the perspectives of children and parents is essential to develop successful ICNs.

## Supplementary Information

Below is the link to the electronic supplementary material.

Supplementary file1 (PDF 291 KB)

Supplementary file2 (PDF 780 KB)

## Data Availability

No datasets were generated or analysed during the current study.
