# Supplementary material for: Integrated care networks in multidisciplinary rehabilitation therapy services for childhood oncology close to home: lessons learned from an international environmental scan
Source: Support Care Cancer. 2025 Apr 23;33(5):406. doi: 10.1007/s00520-025-09421-w (PMC12018495; doi:10.1007/s00520-025-09421-w)
Supplement: Supplementary file 1 — Supplementary file1 (PDF 291 KB) [file 520_2025_9421_MOESM1_ESM.pdf]

Supplementary data I

Journal

Supportive Care in Cancer

Title:

Integrated care networks in multidisciplinary rehabilitation therapy services for childhood oncology close to home: lessons learned from an international environmental scan.

Authors:

L.B. Kleinlugtenbelt [1], PT, PCS, MSc (ORCID: 0000-0001-7782-0910)
J.W. Gorter [2,3] MD, PhD (ORCID: 0000-0002-3012-2119)
E.C. van Dalen [1], MD PhD (ORCID: 0000-0002-8886-6532)
M. Ketelaar [3], PhD (ORCID: 0000-0002-8324-518X)
W.J.E. Tissing [1,4], MD, PhD
Corresponding author: W.J.E. Tissing, w.j.e.tissing@prinsesmaximacentrum.nl

Search strategy for Pubmed/MEDLINE:

1) Childhood cancer:

leukemia OR leukemi* OR leukaemi* OR "childhood ALL" OR AML OR (leukemia, lymphocytic, acute[mh]) OR (leukemia, lymphocytic, acute*) OR lymphoma OR lymphom* OR hodgkin OR hodgkin* OR T-cell OR B-cell OR non-hodgkin OR non-hodgkin* OR sarcoma OR sarcom* OR sarcoma, Ewing's OR Ewing* OR osteosarcoma OR osteosarcom* OR wilms tumor OR wilms* OR nephroblastom* OR neuroblastoma OR neuroblastom* OR rhabdomyosarcoma OR rhabdomyosarcom* OR teratoma OR teratom* OR hepatoma OR hepatom* OR hepatoblastoma OR hepatoblastom* OR PNET OR medulloblastoma OR medulloblastom* OR PNET* OR neuroectodermal tumors, primitive OR retinoblastoma OR retinoblastom* OR meningioma OR meningiom* OR glioma OR gliom* OR brain tumor OR brain tumor* OR brain tumour* OR brain cancer* OR brain neoplasm* OR intracranial neoplasm* OR brain neoplasms OR central nervous system neoplasm OR central nervous system neoplasms OR central nervous system neoplasm* OR central nervous system tumor OR central nervous system tumour OR central nervous system tumor* OR central nervous

system tumour* OR “pediatric oncology” OR “paediatric oncology” OR “childhood cancer” OR “childhood tumor” OR “childhood tumors”

2) Children *(Leclercq E, Leeflang MM, van Dalen EC, Kremer LC. Validation of search filters for identifying pediatric studies in PubMed. J Pediatr. 2013 Mar;162(3):629-634.e2):*

infan* OR newborn* OR new-born* OR perinat* OR neonat* OR baby OR baby* OR babies OR toddler* OR minors OR minors* OR boy OR boys OR boyfriend OR boyhood OR girl* OR kid OR kids OR child OR child* OR children* OR schoolchild* OR schoolchild OR school child[tiab] OR school child*[tiab] OR adolescen* OR juvenil* OR youth* OR teen* OR under*age* OR pubescen* OR pediatrics[mh] OR pediatric* OR paediatric* OR peadiatric* OR school [tiab] OR school*[tiab] OR prematur* OR preterm*

3) Integrated care networks part 1:

Delivery of Health Care, Integrated OR “Integrated Health Care Systems” [tiab] OR “Integrated Health Care System” [tiab] OR “Integrated Delivery Systems” [tiab] OR “Integrated Delivery System” [tiab] OR “Models, Organizational” [tiab] OR “Organizational Models” [tiab] OR “Organizational Model” [tiab] OR organizational model* OR “Organisational Models” [tiab] OR “Organisational Model” [tiab] OR organisational model* OR “Cooperative Behavior” [tiab] OR “Cooperative Behaviors” [tiab] OR cooperative behav* OR “Cooperative Behaviour” [tiab] OR “Cooperative Behaviours” [tiab] OR “integrated care” [tiab] OR “person-integrated care” [tiab] OR “person integrated care” [tiab] OR “shared care” [tiab] OR “Patient care team” [tiab] OR “patient care teams” [tiab] OR “multidisciplinary Care Team” [tiab] OR “Multidisciplinary Care Teams” [tiab] OR “medical care team” [tiab] OR “medical care teams” [tiab] OR “healthcare team” [tiab] OR “Healthcare Teams” [tiab] OR “Interdisciplinary Health Team” [tiab] OR “Interdisciplinary Health Teams” [tiab] OR “Multidisciplinary Health Team” [tiab] OR “Multidisciplinary Health Teams” [tiab] OR “Health Care Team” [tiab] OR “Health Care Teams” [tiab] OR “interprofessional relations” [tiab] OR “interprofessional relation” [tiab] OR “Accountable Care Organizations” [tiab] OR “Accountable Care Organization” [tiab] OR accountable care organi* OR “accountable care organisations” [tiab] OR “accountable care organization” [tiab] OR “patient-centered care” [tiab] OR “patient centered care” [tiab] OR “person-centered care” [tiab] OR “person centered care” [tiab] OR “patient-focused care” [tiab] OR “patient focused care” [tiab] OR “comprehensive health care” [tiab] OR “comprehensive healthcare” [tiab]

4) Integrated care networks part 2:

(integrat* [tiab] OR collaborat* [tiab] OR transmur* [tiab] OR multidisciplin* [tiab] OR interdisciplin* [tiab]) AND care [tiab]

Search: #1 AND #2 AND (#3 OR #4)

Limits: English language
