# Supplementary material for: Integrated care networks in multidisciplinary rehabilitation therapy services for childhood oncology close to home: lessons learned from an international environmental scan
Source: Support Care Cancer. 2025 Apr 23;33(5):406. doi: 10.1007/s00520-025-09421-w (PMC12018495; doi:10.1007/s00520-025-09421-w)
Supplement: Supplementary file 2 — Supplementary file2 (PDF 780 KB) [file 520_2025_9421_MOESM2_ESM.pdf]

Supplementary data II

Journal

Supportive Care in Cancer

Title:

Integrated care networks in multidisciplinary rehabilitation therapy services for childhood oncology close to home: lessons learned from an international environmental scan.

Authors:

L.B. Kleinlugtenbelt [1], PT, PCS, MSc (ORCID: 0000-0001-7782-0910)

J.W. Gorter [2,3] MD, PhD (ORCID: 0000-0002-3012-2119)

E.C. van Dalen [1], MD PhD (ORCID: 0000-0002-8886-6532)

M. Ketelaar [3], PhD (ORCID: 0000-0002-8324-518X)

W.J.E. Tissing [1,4], MD, PhD

Corresponding author: W.J.E. Tissing, w.j.e.tissing@prinsesmaximacentrum.nl

Supplementary data

E-MAIL TO RESPONDENTS, BACKGROUND INFORMATION AND SURVEY QUESTIONS

Dear,

The Princess Maxima Center for childhood oncology in Utrecht, is the national Dutch center in which all Dutch childhood cancer patients are diagnosed and treated. The centralization has made it possible for us to provide top care, conduct top research and provide optimal support for children and parents. And very important: care and research work closely together. The downside of centralization is the often-long distance between the hospital and home. Part of treatment can be done locally (eg physiotherapy, psychology etc), but those professionals are not always optimally equipped for care for these patients with a rare disease. Therefore, we started a project to improve the quality and continuity of care for children with cancer closer to home, during and after treatment. By care we mean care by allied healthcare professionals (psychology, pediatric physical therapy, dieticians, occupational therapy and speech and language therapy).
This project, called KinderOncoNet will contribute in developing a multidisciplinary integrated care network [1-4] with the aim to improve the continuity and quality of care locally. By integrated care networks we mean; a coordinated way of working across multiple professionals, organisations and sectors in order to improve the health, quality of care and economic outcomes for a targeted (sub)population. KinderOncoNet would allow for sharing knowledge, developing skills, and improving accessibility and communication in the Netherlands. See our website KinderOncoNet - Prinses Máxima Centrum (prinsesmaximacentrum.nl), also in english.

In building the network it is important to learn from other initiatives of integrated care networks for childhood oncology. Therefore, we use an environmental scan as a method to systematize knowledge. We contact you because we hope we can learn from your initiatives in coordinating the care of allied health care professionals for children with childhood oncology and survivors close to home. If not, let us now.

We kindly ask you for your cooperation in answering the questions   in the survey below, with the aim to learn from your experiences in coordinating the care of allied health care professionals for children with childhood oncology and survivors close to home. Your help is needed to develop KinderOncoNet as best as possible, so we can build on your experiences.

If you feel are not the right person to answer the survey questions, feel free to send this email to a colleague with more experience in this field.
Please complete the survey by the 22th of April and return by e-mail please.
Or let us know if you prefer to discuss the topics in an online meeting.

Thank you very much for your cooperation. Your name and center will be named in the acknowledgement of the manuscript to be published.
Kind regards, on behalf of the steering group KinderOncoNet,

Wim Tissing, Professor Childhood Oncology and Head of research Supportive Care, Prinses Maxima Center for pediatric Oncology, Utrecht, The Netherlands

Lineke Rehorst-Kleinlugtenbelt, MSc, Pediatric physical therapist and Clinical health scientist
Project leader KinderOncoNet en PhD candidate Supportive Care, Prinses Maxima Center for pediatric Oncology, Utrecht, The Netherlands

Jan Willem Gorter, Professor and Head of Pediatric Rehabilitation, Department of Rehabilitation, Physical Therapy Science and Sports, University Medical Center Utrecht, The Netherlands.

INTRODUCTION/BACKGROUND/RATIONALE

In the Netherlands around 600 children are diagnosed with cancer each year [5]. Since 2018, care for children with cancer in The Netherlands is centralized in the Princess Máxima Center for pediatric oncology in Utrecht. The mission is to cure every child with cancer with optimal quality of life. Our center aims to centralize care when needed and to provide care locally when possible. Parts of the oncological treatment can be administered in one of fifteen Shared Care Centers closer to home, or at home.

The treatment of childhood cancer often comes with many side effects. With higher cure rates, the total number of childhood cancer survivors increases, resulting in increasing numbers of patients with short and long-term medical, physical, and psychosocial needs, often extending into adulthood [6, 7]. The 5-year survival rate after childhood cancer is 80%, with more than 16.000 survivors in The Netherlands. 75% of the survivors have 1 or more late effects of the treatment.

Because childhood cancer is rare, allied health care professionals outside the Princess Máxima Center or shared care center don't often see children during or after their treatment. For example, continuity of care by pediatric physical therapists (PTTs) at home is critically important to reduce side-, and late effects of the oncological disease [8, 9]. Unfortunately, currently we lack a network of specialized pediatric physical therapists (PPTs) in childhood cancer across settings in The Netherlands. Most of the local PPTs in The Netherlands only see none to four children with childhood cancer in their whole career [10]. Thus, in combination with the diversity of cancer types, developmental stages (0-18 years) and various needs, a PPT in the community will almost always lack specialised knowledge and experience.
For physiotherapists, psychologists, dieticians, teachers, but also rehabilitation doctors and other medical specialists, it is important that in addition to existing professional knowledge from the Princess Máxima Center, targeted training and advice is offered. This is even more important since we know that developing an integrated care model is hindered by the lack of continuity of care across settings and the lack of knowledge/expertise/experience in the community [10].
Therefore, to optimize continuity and quality of care by close to home, it makes sense that PPTs need access to up-to-date knowledge and expertise related to pediatric oncology. The same is true for other allied healthcare professionals. Developing an integrated care network of allied health care professionals could be a solution to overcome this care gap.

Worldwide, there is an understanding that the concept of network medicine is critical to meet the needs of a growing population of childhood cancer survivors [11]. Facilitating the right care in the right place is one of the most important objectives of network care. This approach means not only prevention of more expensive care in specialized centers, but also to move care closer to people's homes, whenever possible, for better quality and efficiency [12-14]. Goals of such healthcare networks are often related to a quadruple aim, i.e., improving the patient experience of care, the health of populations, reducing per capita costs and improving the work life of those who deliver care [1].

In building the network it is important to learn from other initiatives of integrated care networks for childhood oncology. Therefore, we use an environmental scan as a method to systematize knowledge. We contact you because we hope we can learn from your initiatives in coordinating the care of allied health care professionals for children with childhood oncology and survivors close to home. If not, let us now.
See our website [KinderOncoNet - Prinses Máxima Centrum (prinsesmaximacentrum.nl)].

We kindly ask you for your cooperation in answering the survey questions below, with the aim to learn from your experiences in coordinating the care of allied health care professionals for children with childhood oncology and survivors close to home. Your help is needed to develop KinderOncoNet as best as possible, so we can build on your experiences.
If you are not the right person to answer the survey questions, feel free to send this email to a colleague with more experience in this field.
Please complete the survey before the 22nd of April and return by e-mail please.
Or let us know if you prefer to discuss the topics in an online meeting.

Thank you very much for your cooperation. Your name and center will be named in the acknowledgement of the manuscript to be published.
Kind regards, on behalf of the steering group KinderOncoNet,

Lineke Rehorst-Kleinlugtenbelt, MSc, Pediatric physical therapist and Clinical health scientist Projectleader KinderOncoNet en PhD candidate Supportive Care, Prinses Maxima Center for pediatric Oncology, Utrecht, The Netherlands

Wim Tissing, Professor Childhood Oncology and Head of research Supportive Care, Prinses Maxima Center for pediatric Oncology, Utrecht, The Netherlands

Jan Willem Gorter, Professor and Head of Pediatric Rehabilitation, Department of Rehabilitation, Physical Therapy Science and Sports, University Medical Center Utrecht, The Netherlands.

SURVEY QUESTIONS

- What is the name of your center/hospital and city name of place of your center/ hospital for childhood oncology?

- How many km is the range in which you treat children with cancer? 0-100km/100-250km/ 250-500km/>500km

- How far away is the next center/ hospital for childhood oncology?
  100km/100-250km/ 250-500km/>500km

- Is there a collaboration agreement with other (shared care) centers/hospitals closer to home?

- How many new patients per year?
  0-100/ 100-250/ 250-500/>500

- Which allied healthcare professionals work in your center?
  Psychologists: Yes/No
  Pediatric physical therapists: Yes/No
  Dieticians: Yes/No
  Occupational therapists: Yes/No

Speech and language therapists: Yes/No
Other:

- Do you have an integrated network for care close to home?
  Yes/No

- Do you see outpatient children in your follow up clinic?
  Yes/No
  If yes also by allied health care professionals?
  If yes, which ones?

- How is integrated care close to home organized in your hospital, for the disciplines, psychology, dieticians, pediatric physical therapy, speech and language therapy and occupational therapy?

- In your experience, what is helping to build/ develop/ implement an integrated care network to improve the care of pediatric oncology patients and childhood cancer survivors close to home?

- In your experience, what is hindering to build/ develop/ implement an integrated care network to improve the care of pediatric oncology patients and childhood cancer survivors close to home

- What is for you the added value of integrated care networks to improve the care in pediatric oncology close to home for your specific center?

- Did you evaluate the integrated care close to home?

- Are there any documents, website information to share about how the integrated care is organized in your country?

- Are there any other initiatives of integrated care you are aware of in your country or outside your country? If yes, which ones?

- Last question, What should an ideal integrated care network look like?
